# *Mentha piperita* Oil Exerts an Antiepileptic Effect in Pilocarpine and Pentylenetetrazol-Induced Seizures in Mice

**DOI:** 10.1155/2022/4431317

**Published:** 2022-09-22

**Authors:** Waleed K. Abdulsahib, Sarmed H. Kathem, Mohanad Y. Al-Radeef, Layth S. Jasim

**Affiliations:** ^1^Department of Pharmacology and Toxicology, College of Pharmacy, Al Farahidi University, Baghdad, Iraq; ^2^Department of Pharmacology and Toxicology, College of Pharmacy, University of Baghdad, Baghdad, Iraq; ^3^Clinical Pharmacy Department, College of Pharmacy, Tikrit University, Tikrit, Iraq; ^4^Department of Chemistry, College of Education, University of Al-Qadisiyah, Diwaniya, Iraq

## Abstract

**Introduction:**

Epilepsy is a progressive, chronic neurological disorder characterized by recurrent seizures. Peppermint (*Mentha piperita* L.) (MP) is one of the most commonly ingested herbal teas or tisanes with a single component.

**Aim:**

We aimed to assess the potential antiepileptic and neuroprotective features of MP essential oil (MPO) in pilocarpine (P) and pentylenetetrazol (PTZ) models of epilepsy.

**Methods:**

The study used eight groups of mice to assess the anticonvulsant activity of MPO in both the P and PTZ acute models in mice. P (350 mg/kg, i.p.) was given 30 minutes after MPO (1.6, 3.2, and 6.4 ml/kg, i.p.). As a positive control group, diazepam (1 mg/kg, i.p) was used. PTZ (95 mg/kg, i.p.) was given 30 minutes after MPO (6.4 ml/kg, i.p.). The first convulsion's latency time, the number of convulsions, the latency time to death, and the percentage of deaths were calculated in all groups.

**Results:**

MPO significantly (*P* < 0.05) increases the first convulsion's latency time and the death's latency time. Moreover, the essential oil significantly decreases the number of convulsions and reduces the mortality rate compared to the negative control group.

**Conclusion:**

MPO at 3.2 and 6.4 ml/kg doses can reduce the percentage and the number of convulsions and increase the latency time of both the first convulsion and death so that it can be used as a supplement in the treatment of epilepsy.

## 1. Introduction

Recurrent seizures describe the progressive, chronic neurological condition known as epilepsy [1]. It is a collection of distinct seizure types and syndromes caused by various processes that share brain neurons' abrupt, excessive, and synchronized firing [2]. In addition to fast head and eye movements, momentary loss of consciousness, spasms, and uncontrolled muscular contractions, various diseases are associated with the manifestation of seizures [2]. Epilepsy affects around 65 million individuals globally, with over 30% being pharmacoresistant to presently available anticonvulsant medicines [3]. Aside from the disease's dismal prognosis, numerous issues (motor problems, anxiety, cognitive deficiencies, depression, and social impairment) also lead to decreased quality of life [4]. Several anticonvulsant medicines are accessible for the pharmacological medication of epilepsy cases across the globe, such as (brivaracetam, carbamazepine, clobazam [5], lamotrigine, phenobarbital, and divalproex). However, seizures remain resistant in over 20% of cases [6]. In addition to failing to control seizures in certain individuals, the existing antiepileptic medicines also have significant side effects [7].

Pentylenetetrazol (PTZ) is a central and respiratory stimulant, similar to doxapram hydrochloride. It is an antagonist of the gamma-aminobutyric acid (GABA) A receptor and is anxiogenic [8]. PTZ induces oxidative stress, increases cortical malondialdehyde content, and affects the hippocampus, resulting in seizures [9]. PTZ is used to produce a model of chemically induced epilepsy. PTZ-induced seizures are characterized as a model of generalized seizures among all animal seizures and epilepsy models (versus partial or focal seizures). It causes petit mal-like or myoclonic seizures [9].

Pilocarpine (P) is a muscarinic agonist that is widely used in many laboratories [10] to induce the status epilepticus (SE) model, which is very similar to human diseases [11]. SE is one of the most frequent medical crises affecting epilepsy cases and is clinically characterized as a seizure lasting more than 5 minutes or repeated seizures within this period without recovery of consciousness [12]. The pilocarpine-caused SE model is normally responsive to most antiseizure medicines when delivered before or concomitantly with a seizure start. Yet, this paradigm is valuable for investigating pharmacological effectiveness in a severe seizure model [13].

Peppermint (*Mentha piperita* L.) is among the most often drunk single-ingredient herbal teas, or tisanes [14]. MPO is well known for its traditional usage in treating fever, digestive, antifungal, cold, oral mucosa, and throat irritation, and for viral infection. Aroma effects are likely caused by a broad range of bioactive phytochemicals, involving flavonoids [15], phenolics, stilbenes, lignans, and essential oils [14]. With the World Health Organization's permission, the medicinal plants' treatment of epilepsy is gaining popularity due to the plants' fewer side effects and diverse beneficial ingredients [16]. Different essential oils (EOs) extracted from various plants have historically been utilized as an alternative therapy for central nervous system illnesses (CNS). Current research focuses on their possible role in neurological illnesses, including stroke, epilepsy, and Alzheimer's, emphasizing their antioxidant [17] and anticonvulsant properties [18].

This research examines MPO's potential antiepileptic and neuroprotective effects in pilocarpine and PTZ epilepsy models. The particular aromatic plants were selected because they are readily available and frequently utilized in aromatherapy and as culinary herbs, investigating and applying their frequent features and possible applications reasonably simple.

## 2. Materials and Methods

### 2.1. Animals

Fifty male and female mice were gathered from the Al-Farahidi University's animal house, College of Pharmacy. The animals were kept in plastic cages in groups of 6 with free access to water and food.

### 2.2. Materials

An insulin syringe cage 31 (PIC Italia) and a balance (SF_400C compact electronic scale, China) were used for IP injection and mouse weighing, respectively.

### 2.3. Chemicals

Diazepam (10 mg/2 ml) sterile pyrogen ampoules were purchased from DEVA, Turkey. Pilocarpine HCl (apicarpine 2%)® sterile eye drops, each 1 ml containing 20 mg of pilocarpine HCL, were obtained from Amman Pharmaceutical Industries Company (Jordan). *Mentha piperita* L. (essential oil) was obtained from Sigma Aldrich (Germany). Phosphate buffered saline with pH 7.4 (9.9 g/L), a product of HIMDIA, INDIA, was used in addition to pentylenetetrazol powder (SIGMA, P6500-25G, India).

### 2.4. Experimental Procedure

Eight groups (6 animals for each) of both sexes, weighing about 25–33 g, were randomly allocated as follows:

Group 1: IP injection of pilocarpine at a dosage of (350 mg/kg). Group 2: IP injection of diazepam with a dose of (1 mg/kg), then after 30 minutes, the animal was injected with (350 mg/kg) pilocarpine (positive group). Group 3: IP injection of (6.4 ml/kg) MPO, then after 30 minutes, injected with (350 mg/kg) pilocarpine. Group 4: IP injection of (3.2 ml/kg) MPO, then after 30 minutes, injected with (350 mg/kg) pilocarpine. Group 5: IP injection with a dose of (1.6 ml/kg) MPO, then after 30 minutes, injected with (350 mg/kg) pilocarpine. Group 6: PTZ's IP administration with a dose of (95 mg/kg). Group 7: IP injection of (1 mg/kg) diazepam, then after 30 minutes, injected with PTZ (95 mg/kg). Group 8: IP injection with a dose of (6.4 ml/kg) MPO, then after 30 minutes, injected with PTZ (95 mg/kg). The onset time, the convulsions' number, as well as the mortality rate were then evaluated in all groups.

## 3. Results

**Figure 1:**
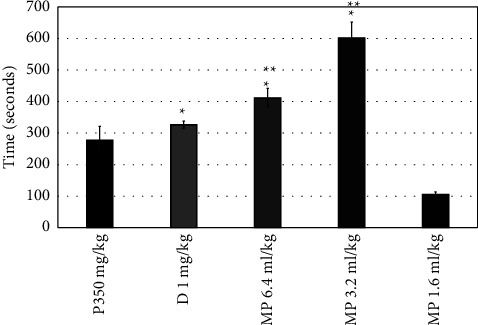
Effects of MPO (6.4, 3.2, and 1.6 ml/kg) on the latency time of the first convulsion in the pilocarpine-induced epilepsy model in mice, ^*∗*^*P* < 0.5 when contrasted to the P350 group, ^*∗∗*^*P* < 0.5 when contrasted to the D group. P: pilocarpine, D: diazepam, MPO: *Mentha piperita L*. oil.

**Figure 2:**
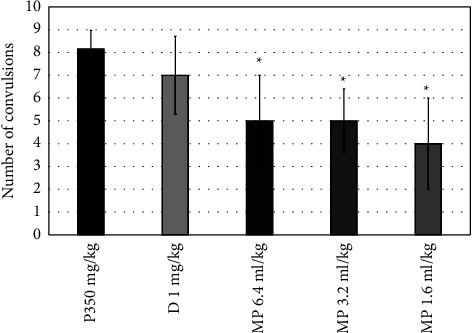
Effects of MPO (6.4, 3.2, and 1.6 ml/kg) on the number of convulsions in the pilocarpine-induced epilepsy model in mice, ^*∗*^*P* < 0.5 when compared to the P350 group.

**Figure 3:**
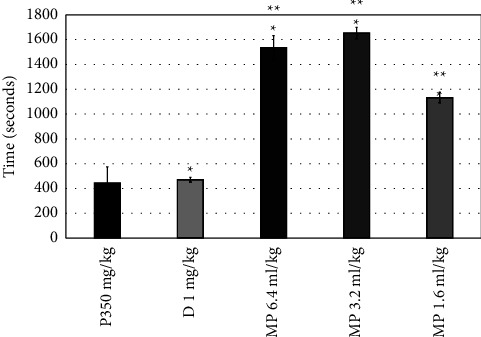
Effects of MPO (6.4, 3.2, and 1.6 ml/kg) on latency time of death in the pilocarpine-induced epilepsy model in mice, ^*∗*^*P* < 0.5 when contrasted to the P350 group, ^*∗∗*^*P* < 0.5 when contrasted to the D group. P: pilocarpine, D: diazepam, MP: *Mentha piperita L*. oil.

**Figure 4:**
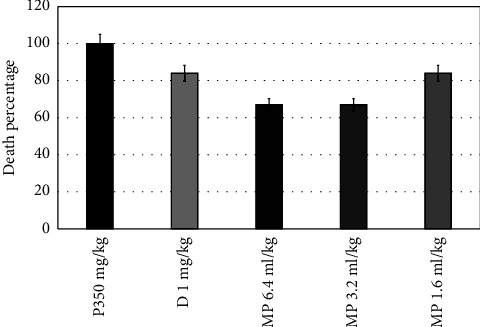
Effects of MPO (6.4,3.2, and 1.6 ml/kg) on death percentage in pilocarpine epilepsy model in mice, ^*∗*^*P* < 0.5 when contrasted to the P350 group, ^*∗∗*^*P* < 0.5 when contrasted to the D group. P: pilocarpine, D: diazepam, MPO: *Mentha piperita L*. oil.

Diazepam increased the latency time to the first convulsion from (277.5 ± 43.5 to 326.5 ± 11.4 seconds) compared to the pilocarpine group (Figure 1) and also significantly increased this time in the PTZ group (*P*=0.0001). All doses of MPO significantly increased the latency time to the first convulsion (*P*=0.0001), as shown in Figure 1. MPO at doses of 1.2, 3.2, and 6.4 ml significantly reduced the convulsions' number (*P*=0.042, 0.002, and 0.042, consecutively) (Figure 2) and increased the latency to death (*P*=0.0001), when compared to the pilocarpine group (Figure 3). Moreover, compared to the diazepam group, MPO significantly increased the latency time to death (*P*=0.0001).  Figure 4 shows that MPO reduced the percentage of deaths to 67% compared to 100% deaths in the induction group.

**Figure 5:**
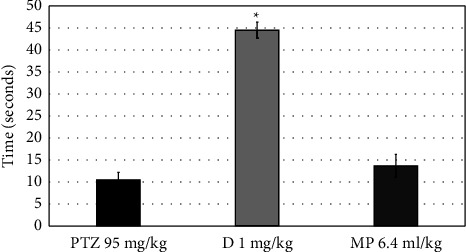
Effects of MPO (6.4 ml/kg) on the latency time to the first convulsion in PTZ-induced epilepsy model in mice, ^*∗*^*P* < 0.5 when contrasted to the PTZ group. PTZ: pentylenetetrazol, D: diazepam, MP: *Mentha piperita L*. oil.

**Figure 6:**
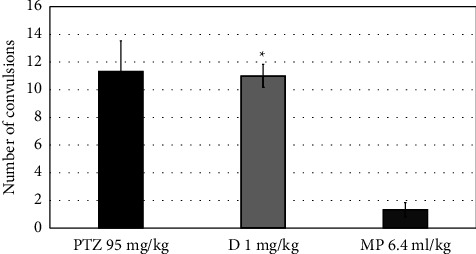
Effects of MPO (6.4 ml/kg) on the number of convulsions in PTZ-induced epilepsy model in mice, ^*∗*^*P* < 0.5 when compared to the PTZ group. PTZ: pentylenetetrazol, D: diazepam, MP: *Mentha piperita L*. oil.

**Figure 7:**
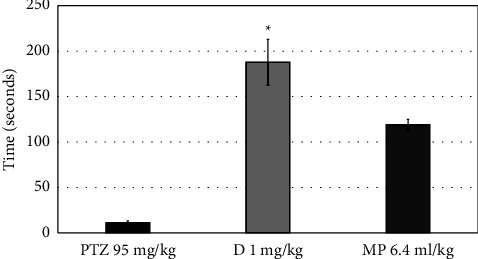
Effects of MPO (6.4 ml/kg) on the latency to death in PTZ-induced epilepsy model in mice, ^*∗*^*P* < 0.5 when compared to the PTZ group. PTZ: pentylenetetrazol, D: diazepam, MP: *Mentha piperita L*. oil.

Contrasted to the PTZ-induced group, MPO (6.4 ml) increased the latency time of the first convulsion but insignificantly (*P*=0.993), as presented in Figure 5.  Figure 6 shows that MPO did not significantly reduce the number of convulsions (*P*=0.967). Furthermore, MPO (at a high dose) increased the latency to death in the PTZ-induced group, but only marginally (*P*=0.195) (Figure 7).

## 4. Discussion

Epilepsy is a condition of the central nervous system featured by frequent seizures caused by the coordinated firing of neuronal networks in the brain [18]. Animal models of epilepsy are exhaustively described to comprehend the illness's neurobiology and identify new antiepileptic compounds [19]. The most prevalent seizure models are produced by pharmacological administration of convulsant drugs (pentylenetetrazol and pilocarpine) or electrical stimulation of specific brain areas at their threshold levels [19]. This approach has been utilized to evaluate neuronal impairment following epileptic episodes since the histological alterations exhibited in the epileptic cases' brains are also present in the brains of chemical-kindled animals [20]. Several studies indicated that seizures cause the generation of reactive oxygen species (ROS) and reactive nitrogen species (RNS) in the brain, which is an organ susceptible to oxidative stress. ROS generation increases cytoplasmic Ca^2+^ concentrations, directly impacting GABA A receptor function, boosting neuronal hyperexcitability, and modifying neuronal membrane possibility [21, 22]. Oxidative stress causes mitochondrial failure, and this dysfunction may induce epileptic seizures via decreased ATP synthesis and altered Na^+^/K^+^ ATPase activity in the cell membrane [21]. Thus, compounds with antioxidant activity [17] may aid in treating epilepsy by lowering oxidative stress in the brain. The most important peppermint components with antioxidant activity are menthol, menthone, neomenthol, methyl acetate, and 1,8 cineole. The study demonstrated that MPO prolonged seizures' onset time and reduced convulsions' frequency and intensity [20]. Our study is in accordance with Zhang et al., who reported that the menthol's administration (200 mg/kg, i.p.) significantly prolonged the clonic and tonic seizures' latency induced by pentylenetetrazol injection. Additionally, menthol significantly lowered the mice's death rate. Consequently, menthol exerts an anticonvulsant impact in the PTZ mouse model, likely via its enhancement of tonic GABAergic inhibition [23].

The peppermint extract prevented mice from experiencing PTZ-induced epileptic seizures and had anticonvulsant properties at a dose of 6.4 ml/kg when given 30 minutes after PTZ injection, producing more favorable results than diazepam. Such therapeutic effects can be attributed to the existing components in the derived extract. For instance, limonene is one of the components of peppermint. Studies have shown that limonene reduces the neurons' simultaneous collective activity associated with the central nervous system (CNS). It enters the brain through peripheral circulation and binds to GABA A receptors, whose activation results in anxiolytic effects [24].

Among the other components of peppermint, linalool has a suppressive effect rooted in inhibiting acetylcholine release [25]. Linalool is a competitive N-methyl-D-aspartate (NMDA) receptor antagonist, and its antiepileptic actions are enhanced by blocking the glutamate NMDA receptor [26]. The anticonvulsant properties of menthone, as another component of peppermint extract, have been demonstrated in previous studies by Jain et al. 2011, who reported that in electroshock or subcutaneous PTZ-induced seizure model, 3-menthone prevented seizures by elevating GABA levels in the midbrain [27]. Administration of essential oils delays the onset of PTZ-induced seizures and reduces their severity in mice [28]. Hangar et al. demonstrate that *Melissa officinalis* L., a member of the Lamiaceae family, has neuroprotective activity in the hippocampus by preventing pilocarpine-induced neuronal loss [29]. Oksana and Michael also demonstrated the antiepileptic effects of Lamiaceae family herbs such as *Scutellaria lateriflora* (Skullcap), *Gelsemium sempervirens* (Gelsemium), and *Datura stramonium* (Jimson Weed) in a lithium-pilocarpine rat model [30]. All these previous studies support that the active constituent of MPO reduces the number of convulsions, increases the latency time of convulsions, and reduces the percentage of deaths in mice.

## 5. Conclusion

MPO oil at 3.2 and 6.4 ml/kg doses can reduce the percentage and number of convulsions and increase the latency time to both the first convulsion and death so that it can be used as a supplement in treating epilepsy.

## Data Availability

All data are available upon request through waleedkalel22@yahoo.com.
